# Impaired Adaptation and Laminar Processing of the Oddball Paradigm in the Primary Visual Cortex of Fmr1 KO Mouse

**DOI:** 10.3389/fncel.2021.668230

**Published:** 2021-05-19

**Authors:** Alexandr Pak, Samuel T. Kissinger, Alexander A. Chubykin

**Affiliations:** Department of Biological Sciences, College of Science, Purdue Institute for Integrative Neuroscience, Purdue University, West Lafayette, IN, United States

**Keywords:** adaptation, Fmr1 KO, mismatch, oddball, spatial frequency, V1

## Abstract

Both adaptation and novelty detection are an integral part of sensory processing. Recent animal oddball studies have advanced our understanding of circuitry underlying contextual processing in early sensory areas. However, it is unclear how adaptation and mismatch (MM) responses depend on the tuning properties of neurons and their laminar position. Furthermore, given that reduced habituation and sensory overload are among the hallmarks of altered sensory perception in autism, we investigated how oddball processing might be altered in a mouse model of fragile X syndrome (FX). Using silicon probe recordings and a novel spatial frequency (SF) oddball paradigm, we discovered that FX mice show reduced adaptation and enhanced MM responses compared to control animals. Specifically, we found that adaptation is primarily restricted to neurons with preferred oddball SF in FX compared to WT mice. Mismatch responses, on the other hand, are enriched in the superficial layers of WT animals but are present throughout lamina in FX animals. Last, we observed altered neural dynamics in FX mice in response to stimulus omissions. Taken together, we demonstrated that reduced feature adaptation coexists with impaired laminar processing of oddball responses, which might contribute to altered sensory perception in FX syndrome and autism.

## Introduction

Fragile X Syndrome (FX) is the most common cause of intellectual disability and the inherited form of autism. Nearly 1 in 4,000 males and half as many females are affected by this condition. It is associated with social communication deficits, hyperactivity, and sensory hypersensitivity (Freund and Reiss, 1991). Given the comorbidity of FX and autism, *Fmr1* KO mice (FX mice) represent a well-defined genetic model that can provide neural circuit-level insights into autism, especially considering the vast diversity of phenotypes and manifestations observed in autism spectrum disorders (ASDs). Such diverse alterations posit a challenge to develop effective diagnostic and treatment tools. FX mice have been shown to exhibit cellular, circuit, and behavioral alterations that recapitulate some of the manifestations observed in human individuals with FX. Prior autism research has been mostly focused on social-cognitive and behavioral impairments (Robertson and Baron-Cohen, 2017). However, a recent revision of diagnostic criteria for autism recognized sensory processing as an important factor to be considered (American Psychiatric Association, 2013). Previous research in humans suggests that sensory alterations may be predictive of social communication deficits later in life in autism (Boyd et al., 2010; Turner-Brown et al., 2012).

Both human and animal studies provide evidence that there is impaired information processing in early sensory areas in both FX and autism (Goel et al., 2018; Rais et al., 2018). Sensory hypersensitivity and reduced adaptation to sensory stimuli are some of the hallmark perceptual impairments in autism. An increase in visual detail processing is often reported in this condition. Visual oddball paradigm studies revealed reduced habituation to repeated stimuli and novel distractors in autistic patients (Sokhadze et al., 2017). Similarly, alterations in the event-related potentials during the auditory and visual oddball tasks were found in FX patients (Van Der Molen et al., 2012). Recent work in FX mice found circuit-level impairments in early visual processing, including reduced orientation tuning and functional output from fast-spiking neurons in V1. Reduced orientation tuning of the neurons in the visual cortex correlated with the decreased ability to resolve different orientations of sinusoidal grating stimuli in both mice and human individuals with FX (Goel et al., 2018). Furthermore, altered dendritic spine function and integration were found in layer 4 of the somatosensory cortex in FX mice (Booker et al., 2019). Structural and functional imaging studies of FX mice revealed local hyperconnectivity and long-range hypoconnectivity in V1 (Haberl et al., 2015). Our group has recently shown that there are impaired visual experience-dependent oscillations and altered functional laminar connectivity in V1 of FX mice (Kissinger et al., 2020). Overall, these studies suggest that there may be circuit-level impairments in early sensory processing in FX.

To shed light on the neural basis of atypical visual perception in FX, we investigated how statistical context influences visual information processing by testing both basic and contextual processing of spatial frequencies (SF) in V1 of FX mice. We measured visually evoked potentials (VEPs) and unit responses in an SF oddball paradigm (Ulanovsky et al., 2003; Hamm and Yuste, 2016). Two stimuli were presented at different probabilities so that one was a standard stimulus (STD) (STD, frequent, redundant), which builds a statistical context. Another one was rare and violated the expectations of the STD stimulus leading to a mismatch (MM) response. This response is hypothesized to reflect a perceptual deviance or change detection. First observed in EEG studies in humans as a delayed negative deflection in event-related potentials, later called mismatch negativity (MMN; Naatanen et al., 1978), it has been replicated in different species and sensory modalities (Chen et al., 2015; Musall et al., 2015; Parras et al., 2017). A decrease in the neural response to the standard stimulus (STD), termed stimulus-specific adaptation (SSA), may be attributed to the predictability of the stimulus because the incoming sensory input matches prediction. Alternatively, it may also be explained by the presynaptic short-term plasticity mechanisms. We computed SSA as the difference between control (CTR) and STD (Hamm and Yuste, 2016; Parras et al., 2017). Given that STD and deviant (DEV) stimuli share the same SF, mismatch (MM) response reports moment-to-moment change detection under the high adaptation level in the local microcircuit, so that any response enhancement can be attributed to change detection. MM, similarly to human MMN, was quantified as the difference between DEV and STD stimuli.

Our SF oddball paradigm is different from the prior oddball studies because both STD and DEV stimuli are of the same low-level feature, a spatial frequency (SF) so that they only differ in the global pattern. Prior studies used two stimuli that differed in low-level features (e.g., orientation, frequency) and thus needed a reverse sequence (flip-flop), in which low and high probability stimuli switch to control for feature preference of the neurons. Our oddball paradigm allowed us to investigate how contextual processing depended on neuronal tuning. Specifically, we investigated how oddball responses changed as a function of the neuron’s preferred SF. Furthermore, we investigated how oddball responses are represented by different cortical layers and neuronal types (regular vs. fast-spiking) neurons in WT vs. FX mice.

Here, we performed silicon probe recordings in WT and FX mouse V1 during the SF oddball paradigm. First, we report excessive processing of high SF stimuli in late neural responses. Second, we demonstrate that adaptation is mostly confined to neurons preferring the SF within one octave of the oddball SF in FX, but not in WT mice, in which it spreads beyond that range. Third, mismatch responses were differentially modulated by cortical layers in WT but not in FX mice. Last, we observed altered neural dynamics during the omission paradigm in FX animals.

## Materials and Methods

### Experimental Animals

All animal experiments were approved by the Purdue University Animal Care and Use Committee. The following strains were used to generate mice for this study: B6.129P2-Fmr1tm1Cgr/J (Fmr1 KO, JAX Stock No. 003025), B6.Cg-453 Tg(Thy1-COP4/EYFP)18Gfng/J (Thy1-ChR2-YFP, JAX Stock No. 007612), and wild type (WT) C57/BL6. We used 10 male Fmr1 KO and seven littermate controls. We also bred Thy1-ChR2 with Fmr1 KO mice to generate Thy1-Fmr1 KO mice. We used four male Thy1-Fmr1 KO and four littermate controls. Additionally, we had six male WT mice. In total, we used 14 Fmr1 KO and 17 control animals for physiology experiments. Animals were group-housed on a 12 h light/dark cycle with full water and food access.

### Surgical Procedures

Animal surgeries were performed as previously described (Pak et al., 2020). Briefly, about 2-month-old animals were induced with 5% isoflurane and secured to a motorized stereotaxic apparatus (Neurostar). Their body temperature was controlled using a heating pad, and they were maintained at 1.5–2% isoflurane anesthesia. The scull was exposed to install a small head post and a reference pin. The binocular V1 coordinates (from lambda AP 0.8 mm, LM: ±3.2 mm) were labeled using a Neurostar software with an integrated mouse brain atlas. Medical grade Metabond^TM^ was then used to seal all exposed areas and form a head cap. After surgery, all animals were monitored for 3 days for any signs of distress or infection. Mice were then habituated to a head-fixation apparatus for at least 4 days 90 min per day. They were positioned in front of the monitor that displayed a gray screen. On the recording day, a small craniotomy was made above V1 on one of the hemispheres under 1.5% isoflurane anesthesia. They were then moved to the recording room and head-fixed to the apparatus in front of the monitor screen.

### Electrophysiology

All recordings were performed in awake head-fixed mice. After mice were transferred to the recording room, we inserted a 64-channel silicon probe (Shobe et al., 2015a; channel separation: vertical 25 μm, horizontal 20 μm, three columns, 1.05 mm in length) to perform acute extracellular electrophysiology. Thirty minutes was allowed after insertion for the probe to settle down. Each mouse underwent a maximum of two recording sessions (one per hemisphere). We acquired data at 30 kHz using OpenEphys hardware and software. We used an Arduino board to synchronize recordings and visual stimulus presentations using TTL communication. Custom written Python scripts using PsychoPy and pyserial were used to present visual stimuli and send TTL signals. Trypsin (2.5%) was used to clean the probe after recording sessions.

### Histology

Animals were anesthetized with 100 mg/kg ketamine and 16 mg/kg xylazine solution. Mice were then perfused transcardially with a 1×PBS followed by a 4% paraformaldehyde. After decapitation, their brain was extracted and stored in PFA in the fridge. After 24 h, the brain was sliced in 0.1 mm sections in PBS using a vibratome. Coronal slices were mounted on slides using n-propyl-gallate media and sealed with transparent nail polish. Slices were imaged using light microscopy (VWR) to verify the probe placement in V1.

### Visual Stimulation

We used a PsychoPy, an open-source Python software, to create and present all visual stimulations (Peirce, 2009). A gamma calibrated LCD monitor (22" ViewSonic VX2252, 60 Hz) was used to present visual stimuli. The mean luminance of the monitor was 30 cd/m^2^. The monitor was placed 17 cm in front of the mouse to binocularly present stimuli. To generate visual stimulations for a spatial frequency tuning and an oddball paradigm, we performed a spatial frequency filtering of random noise. Specifically, we bandpass filtered random noise in different non-overlapping SF bands. This was done by performing the following steps. First, we randomly generated noise and converted it to a frequency domain using FFT (numpy FFT). Second, we created a spatial frequency bandpass filter using the Psychopy Butterworth filter with an order of 10. Third, we multiplied the white noise in the frequency domain by our bandpass filter. This step filtered all the frequencies but the desired SF band. Fourth, we took the inverse Fourier transform of our altered frequency domain. The procedure and a Python code for spatial frequency filtering were adapted from http://www.djmannion.net/psych_programming/vision/sf_filt/sf_filt.html. We modified the above code to generate SF filtered noise. Overall, we used six different spatial frequencies for SF tuning: 7.5E-3, 0.015, 0.03, 0.06, 0.12, and 0.24 cycles/degrees. We chose these frequencies based on previous studies and known spatial frequency tuning of mouse V1 neurons. We verified that we could obtain reliable SF tuning similarly to our previous study (Kissinger et al., 2018). SF tuning sequence contained six different SF stimuli presented in a pseudorandom order at equal probability. Each SF was repeated 20 times so that the experiment had 120 trials in total. We used an inter-trial interval of at least 4 s to prevent any adaptation. Furthermore, SF filtered stimuli were randomly generated on each trial to uniformly sample different receptive fields. This was mainly important for lower spatial frequencies. For the oddball paradigm, we used two stimuli of the same SF but different overall patterns. The first stimulus was a standard (STD) with a probability of 0.875. Its texture did not change across trials. The second one was a deviant (DEV) with a probability of 0.125, its overall pattern changed across trials. This was done to maximize the surprise response. Inter stimulus interval was 0.5 s plus a random delay chosen from the range of 0.5 and 1.2 s. The stimulus was presented for 0.5 s. In total, 200 trials were presented during the oddball paradigm. For the omission paradigm, every eighth stimulus was omitted to investigate omission responses. Inter stimulus interval was set to 1.7 s, and 200 trials were presented. Overall, a maximum of 520 trials was presented to a mouse during a single recording session.

### LFP Analysis

Raw electrophysiology traces were first downsampled to 1 kHz. We then used a symmetric linear-phase FIR filter (default parameters) from the mne Python library to remove 60 Hz noise. Next, we identified Layer 4 by finding a channel with the strongest negative deflection in the first 100 ms after stimulus onset. Time-frequency analysis was done using a complex wavelet convolution. Forty different wavelets were designed across a logarithmic range of 2–80 Hz, with cycles ranging from 3 to 10. This gave us an optimal time-frequency precision tradeoff. We convolved these wavelets with averaged LFP traces and then averaged the resulting power spectra across different conditions. For heatmaps, power was dB baseline normalized. To quantify a mean power within a particular band, we averaged responses within a 0.05–0.5 s time window. We used six different frequency bands: theta (4–8 Hz), alpha (8–12 Hz), beta (12–30 Hz), low gamma (30–50 Hz), and high gamma (50–80 Hz).

### Single Unit Analysis

Clustering and manual curation of units were performed as previously described (Pak et al., 2020). Kilosort was used for spike detection and sorting. It uses a template matching algorithm and allows a GPU acceleration (Pachitariu et al., 2016). Default configuration parameters were used for clustering, but a threshold for spike detection was changed from −4 to −6. SD. Templates were initialized from the data. Kilosort was run using MATLAB (Mathworks) on Windows 10 running computer. For clustering purposes, all the different recording blocks were concatenated together. This allowed us to track single neurons across different recording sessions. After clustering, we visualized and verified clustering results using Klusta/Phy GUI. It speeds up the process of manually removing, splitting, and merging units (Rossant et al., 2016). We used several criteria to only include well-isolated units: (1) had more than 10 spikes for each experimental block; (2) less than 5% of spikes violated an absolute refractory period; (3) clean template shape; and (4) templates were localized within a small channel group. To merge and split units, we followed the guidelines available online (https://github.com/kwikteam/phy-contrib/blob/master/docs/template-gui.md). Peristimulus time histograms (PSTHs) of single units were constructed by binning spike times across trials with 10 ms bins and convolving the obtained histogram with a Gaussian Kernel (width = 100 ms). *Z*-score was calculated by the following formula:

$$z=\frac{R-mean(baseFR)}{sd(baseFR)}$$

where, FR is a firing rate at each time point, and base refers to the baseline activity over 0–0.3 s.

For spatial frequency analysis, we averaged the firing rate within 0.05–0.2 s for tuning analysis and 0.2–0.5 s to investigate later responses. Population tuning curves were constructed using baseline-subtracted firing rates across different neurons. We fitted a difference of Gaussian function to SF tuning curves (Hawken and Parker, 1987):

$$R(SF)=R_{0}+K_{e}e^{\frac{-{\left(SF-\mu_{e}\right)}^{2}}{2\sigma_{e}^{2}}}-K_{i}e^{\frac{-{\left(SF-\mu_{i}\right)}^{2}}{2\sigma_{i}^{2}}}$$

This function has seven free parameters: baseline firing rate *R*_0_, amplitude *K*_e_, *K*_i_, center μ_e_ and μ_i_, width σ_e_ and σ_i_ of the excitatory and inhibitory components, respectively.

$$fit\;error=\frac{\sum {\left(y_{i}-f_{i}\right)}^{2}}{\sum {\left(y_{i}-\bar{y}\right)}^{2}}$$

where, *y_i_* is the observed value, $\bar{y}$ is the mean of observed data, and *f_i_* is the fitted value. The fitting procedure was performed using curve_fit from Python. Initial value for each parameter was set to 0.01. Bounds were set to [0, 1] for width and [0, max firing*2] for other parameters. Tuning sharpness was quantified using the quality factor (Q):

$$Q=\frac{SF_{peak}}{SF_{high}-SF_{low}}$$

where *SF_peak_* is the preferred SF of the unit, *SF_high_* and *SF_low_* are the high and low SF cut-offs at which the tuning curve drops below peak $\sqrt{2}$ (Bredfeldt and Ringach, 2002).

To investigate oddball responses, we focused on neurons that upregulate their firing in response to visual stimuli. We used Wilcoxon signed-rank test to identify these neurons by comparing baseline firing rate −0.25–0.05 s vs. stimulus window 0.05–0.35 s. The response to the SF0.03 was used as the control for the oddball paradigm. To equalize the number of trials between STD and DEV stimuli, we only used pre-DEV trials for STD. We computed modulation indices for mismatch response (MM) and stimulus-specific adaptation (SSA) using the following formulas.

$$iSSA=\frac{CTR-STD}{CTR+STD};\;iMM=\frac{DEV_{late}-STD_{late}}{Dev_{late}+STD_{late}}$$

where STD/CTR represents baseline-corrected mean firing rate within 0.05–0.5 s, and STD_late_/DEV_late_ 0.2–0.5 s relative to the stimulus onset.

To investigate how SSA and MM change as a function of preferred SF of the units, we split neurons into three groups: tuned_in, tuned_out, and untuned units. Tuned_in group included units with preferred SF that lies within 1 octave of oddball SF, 0.03 cpd (0.015 < pref SF < 0.06). The tuned_out group included units with preferred SF that lies outside the 1 octave of the oddball SF (pref SF < 0.015 or pref SF > 0.06). The untuned group included units that did not show any SF tuning properties; the fitting procedure was not successful, or the fitting error exceeded 0.9. These units were then further split by the cortical depth. The layer of each neuron was assigned based on the depth of the channel with the strongest negative deflection of the template. We used Kilosort template waveform features to split units into putative regular or fast-spiking (RS vs. FS) neurons. FS units were defined as those with trough-to-peak times less than 0.45 and spike width less than 1.2. RS units, on the other hand, had trough-to-peak times more than 0.45 and spike width larger than 1.2. Units that fall in between were defined as unclassified.

The omission paradigm was analyzed in two different ways. First, we decided to investigate the laminar processing of omission responses. Omission-responsive units were defined as those with significant neural responses during omission (expected stimulus timing vs. baseline 0.05–0.35 vs. −0.25–0.05). Neurons with significant responses were further subdivided into omis-excited and omis-inhibited depending on whether their mean response exceeds 0 or not. Overall, 122 WT and 95 FX units were omis-excited, 93 and 92 omis-inhibited, and 230 WT and 134 FX units did not have a significant omission response. The second approach employed an unsupervised clustering algorithm, *k*-means. The input was omission responses (0.05–0.5 s) from both genotypes. We used scikit-learn implementation of *k-means* and initialized it with PCA for consistency. The number of clusters was determined using an “elbow method,” in which distortion and inertia can be plotted against the number of clusters. It is challenging to find an optimal number of groups for *k-means* with neurophysiology data; however, we observed that *k* = 4 is the point at which a slope changes in the inertia and distortion plots. In addition, we qualitatively observed that four groups captured the diversity of omission responses. Given that genotype of units is independent of the clustering process, we compared omission responses within each *k-means* group.

SF neural decoding was performed using Linear Discriminant Analysis in Python scikit-learn package (default parameters; Virtanen et al., 2020). Population spike counts from different time windows were used to train classifiers. We used 4-fold cross-validation with five repeats. The number of folds was chosen so that the test size was not below 30 samples. We also trained logistic regression (multinomial) and SVM (with RBF kernel) classifiers (data not shown), but LDA gave better performance given the number of parameters to specify. The number of units used for training was comparable in both groups. For example, decoding from the 0.35 to 0.45 s interval was performed using 1,324 units from WT and 1,226 units from FX.

### Statistical Analysis

We used scipy.stats Python library to perform statistical analysis. Data were not tested for normality of residuals, and only non-parametric tests were used. Mann–Whitney *U* test was used to compare two independent populations. It was used to compare a trial-averaged LFP and neuronal firing rate in response. *P-values* were adjusted using a Benjamini–Hochberg procedure that controls for a false discovery rate. Kolmogorov–Smirnov 2 sample test was used to compare distributions of iSSA and iMM indices between WT and FX mice in different layers.

## Results

### Enhanced Oddball Responses in LFP of FX Mice

**Figure 1:**
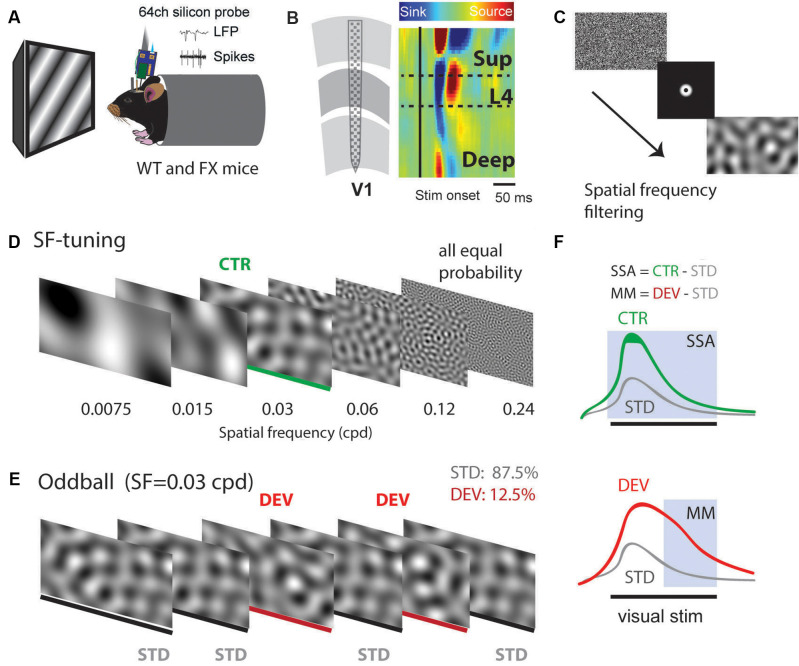
A visual oddball paradigm with all the stimuli containing the same low-level features [spatial frequency (SF)] but different global SF patterns and expectancy. **(A)** *In vivo* extracellular silicon probe recordings in V1 of head-fixed mice. **(B)** Schematic of a 64-channel silicon probe spanning the whole cortical depth and an example of current source density (CSD) heatmap. **(C)** To generate visual stimuli, we performed SF filtering of white noise. **(D)** We used six different non-overlapping SF bands from 7.5E-3 to 0.24 cpd for spatial frequency tuning (many standards control). Stimuli were presented in a pseudorandom order and had equal probability. **(E)** The oddball sequence contained stimuli of the same SF (0.03 cpd) that only differ in their probability and overall texture. Standard (STD) and deviant (DEV) stimuli were presented with a probability of 0.875 and 0.125, respectively. **(F)** Given that STD and DEV have the same low-level features (SF), we computed a neuronal mismatch (MM) response by comparing late (0.3–0.5 s) responses of STD and DEV. Stimulus-specific adaptation (SSA) was obtained by comparing STD and CTR. Since both STD and DEV had the same SF, neural population activity is expected to be adapted during the oddball.

Using 64 channel silicon probes that span the cortical depth of V1 (Shobe et al., 2015b), we investigated visual processing of spatial frequencies (SF) during tuning (many standards control) and oddball paradigm in awake head-fixed WT and FX mice (Figures 1A,B). For SF tuning, we presented animals with SF filtered visual noise stimuli using six different non-overlapping SF bands (Figures 1C,D). Stimuli of the same band have the same spatial frequency but a different overall global pattern. These stimuli have been previously validated for tuning measurements. Furthermore, there was no significant difference between WT and FX mice in neural response variability to the same SF band with different overall patterns (Supplementary Figure 1). Oddball responses were analyzed by comparing responses to standard (STD) and control (CTR) stimuli for SSA and delayed part of STD and deviant (DEV) responses for calculating the mismatch (MM) response (Figures 1E,F). In contrast to previous animal oddball studies, our STD and DEV have the same low-level features (SF), so that increased delayed part of the DEV response can be attributed to change detection.

**Figure 2:**
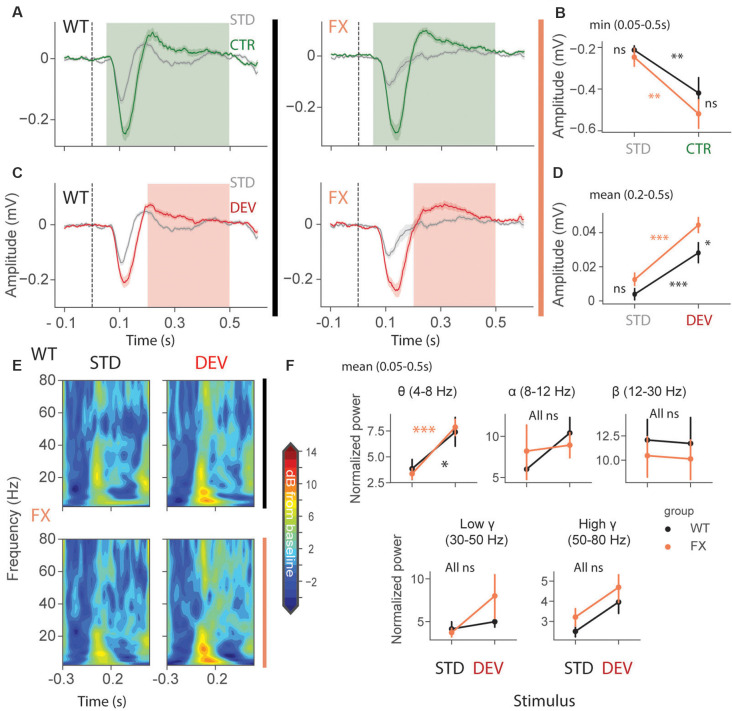
Enhanced late responses in L4 of FX mice during a visual oddball paradigm. **(A)** Averaged layer 4 LFP traces in response to STD and CTR stimuli for WT (left) and FX (right) from cortical layer 4. **(B)** The point plots show the mean and s.e.m. of the strongest negative deflection within 0.05–0.5 s relative to the stimulus onset. **(C)** Same as in **(A)** but comparing STD vs. DEV. **(D)** Same as in **(B)**, but responses were averaged within 0.2–0.5 s. **(E)** Time-frequency spectra of the L4 LFP traces of WT (top) and FX (bottom). **(F)** Point plots show the mean power within 0.05–0.5 s relative to the stimulus onset across different frequency bands. *p < 0.05, **p < 0.01, ***p < 0.001, ns = not significant.

We first focused on oddball responses in local field potential (LFP), which represents local population subthreshold activities. We found adaptation and mismatch responses in layer 4 LFP of both genotypes (Figures 2A,C). Interestingly, MM responses but not SSA were stronger in FX animals [Figures 2B,D, SSA: STD vs. CTR WT (*P* = 0.0057), FX (0.002); WT vs. FX STD (*P* = 0.440), CTR (*P* = 0.105); MM: STD vs. DEV WT (*P* = 0.0016), FX (*P* = 0.0002), WT vs. FX STD (0.075), DEV (*P* = 0.015), *n* = 17 and 15 mice, Mann–Whitney *U* test, *p*-values were adjusted for multiple comparisons using the Benjamini–Hochberg method]. Time-frequency analysis was then performed on L4 LFP to investigate whether any frequency bands are modulated by oddball responses (Figure 2E). Entire duration of DEV response was used, so that the window is big enough to quantify low frequency oscillations. We found that only theta oscillations were modulated by the oddball responses in both genotypes [Figure 2F, STD vs. DEV: theta WT (*P* = 0.021) and FX (*P* = 0.0006); alpha WT (*P* = 0.089) and FX (*P* = 0.089); beta WT (*P* = 0.45) and FX (*P* = 0.45); low gamma WT (*P* = 0.21 and FX (*P* = 0.40), high gamma WT (*P* = 0.05) and FX (*P* = 0.05); WT vs. FX STD and DEV all bands (*P* > 0.05), *n* = 17 WT and 15 FX mice, Mann–Whitney *U* test, *p-values* were adjusted for multiple comparisons within each frequency band using the Benjamini–Hochberg method].

### Excessive Processing of High Spatial Frequencies in V1 of FX Mice in Late Unit Responses

**Figure 3:**
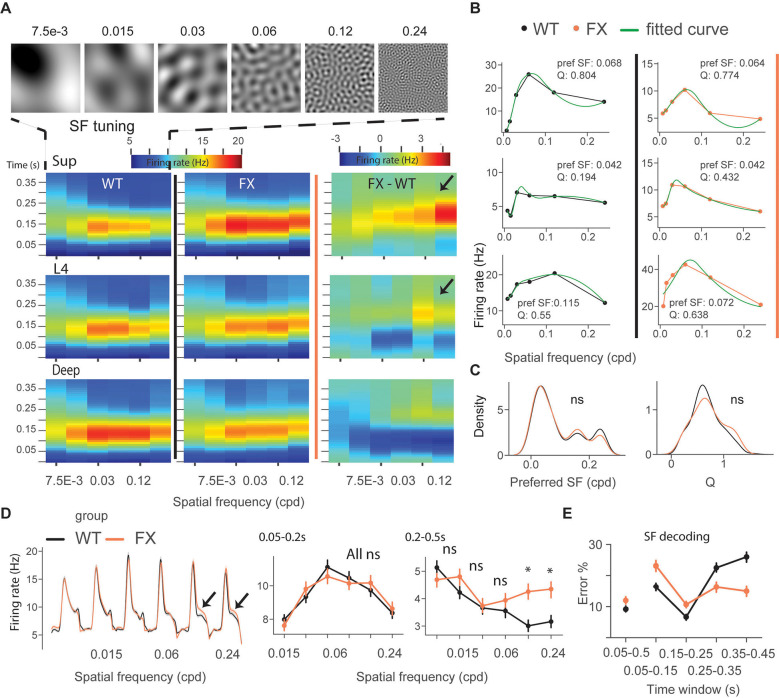
Excessive processing of high SF stimuli in late responses of single units in FX mice.** (A)** Time-course analysis of SF tuning across the cortical layers. Unit responses for different SF stimuli were plotted for each time step to create the heatmaps for WT (left), FX (middle), and FX-WT (right). **(B)** SF tuning curves were computed by averaging responses within 0.05–0.2 s relative to the stimulus onset and fitted using Difference-of-Gaussians. Example plots are shown for WT (left) and FX mice (right). **(C)** Distribution of preferred SF (left) and Q-factor (right) for both groups. The larger Q values indicate sharper tuning. **(D)** Population average firing rates of all units in response to the SF tuning sequence. Note enhanced late part responses at higher SF. The population mean SF tuning responses were averaged for different time intervals. 0.05–0.2 (left) and 0.2–0.5 s (right). **(E)** Population spike counts from different time windows were used for SF neural decoding. The classifiers that were trained on responses after 0.25 s relative to stimulus onset had a lower error in FX vs. WT mice. *p < 0.05, ns = not significant.

We next focused on single-unit activity during tuning (control) and oddball sequence. The time course heatmap of SF tuning revealed enhanced activity in late unit responses in all layers of FX animals, especially at higher SF (Figure 3A). To obtain a preferred SF for each unit, we fitted a Difference-of-Gaussian model to tuning curves, which were obtained by averaging the firing rate within 0.05–0.2 s relative to the stimulus onset (Figure 3B). We did not observe any differences in the distribution of preferred SF or Q-factor (tuning sharpness) between genotypes (Figure 3C) WT vs. FX pref SF (*P* = 0.357), *n* = 949 and 705 units; Q-factor (*P* = 0.404), *n* = 192 and 126 units, Kolmogorov–Smirnov 2 sample test). The population mean responses to different SF stimuli revealed enhanced activity in late unit responses at high SF (Figure 3D). To quantify these differences, we averaged firing rates within different time windows: 0.05–0.2 s for early and 0.2–0.5 s for late visual responses. We found a significantly stronger response at higher SF (>0.06 cpd) in late visual responses [Figure 3D right, WT vs. FX 0.05–0.2 s all stimuli (*P* > 0.05), 0.2–0.5 s: SF 7.5e-3–0.06 (*P* > 0.05), SF 0.12 (*P* = 0.014), and SF 0.24 (*P* = 0.035), *n* = 1,057 and 820 units, Mann–Whitney *U* test, *p*-values were adjusted for multiple comparisons using the Benjamini–Hochberg method].

Next, SF neural decoding was performed using population spike counts (Figure 3E). We reasoned that enhanced processing of higher SF might lead to enhanced detection of these stimuli in FX mice. Classifiers were trained on spike counts from different time windows of WT and FX mice using a linear discriminant analysis with 4-fold cross-validation with five repeats. Classifiers trained on spike counts from 0.05–0.5 s performed similarly (SF classification mean ± SEM % error WT vs. FX: 9.1 ± 0.9 vs. 12.0 ± 1). WT classifiers performed slightly better in early time windows (SF classification mean ± SEM % error WT vs. FX 0.05–0.15 s: 16.3 ± 1.1 vs. 23.1 ± 1.8; 0.15–0.25 s: 6.7 ± 0.9 vs 10.7 ± 1.1). However, classifiers trained on the intervals after 0.25 s show a reduced error in FX vs. WT mice (SF classification mean ± SEM % error WT vs. FX 0.25–0.35 s: 22.5 ± 1.4 vs. 16.3 ± 1.7; 0.35–0.45 s: 26.0 ± 1.6 vs. 15.0 ± 1.6), suggesting enhanced processing in late neural responses. Together, these findings suggest an enhancement of processing in late neural responses in FX vs. WT mice, especially at high spatial frequencies.

### Both SSA and MM Are Present in SF Tuned Units

**Figure 4:**
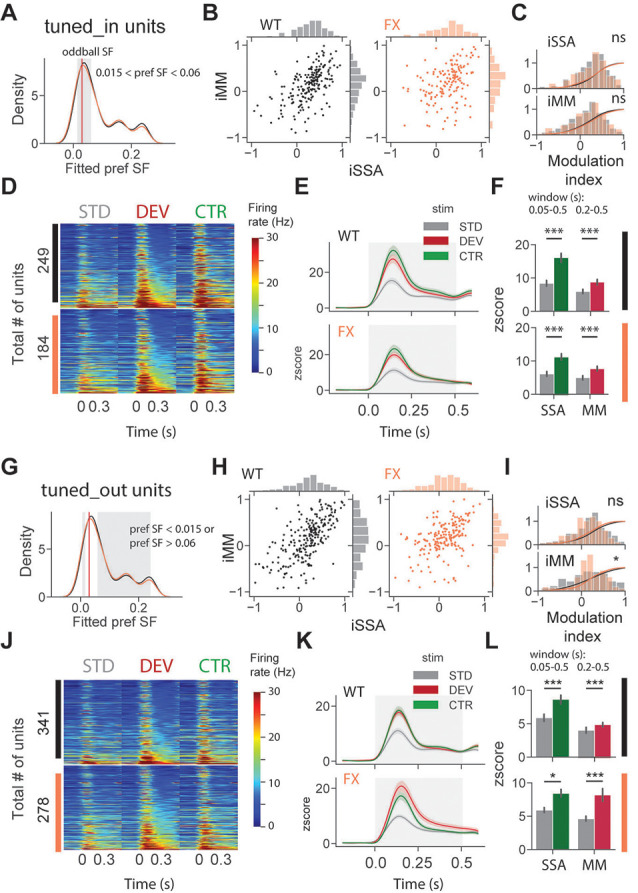
SSA and MM are present in single units tuned to various spatial frequencies of both genotypes. **(A)** Distribution of preferred SF of two different genotypes. Gray shaded area represents tuned_in group, which included units with preferred SF that lies ±1 octave of oddball SF (0.03, red vertical line). **(B)** Distribution of iSSA and iMM modulation indices for WT and FX mice (each point is a single unit). **(C)** Superimposed distributions of iSSA and iMM with KDE. **(D)** The heatmaps show single-unit firing rates in response to STD, DEV, and CTR stimuli across different genotypes. (**E**) The line plots show the mean *z*-scored responses of the units from the heatmaps. **(F)** The point plots show the mean ± SEM of the *z-scored* firing rate between 0.05 and 0.5 s for SSA and 0.2–0.5 s for MM relative to the stimulus onset.** (G–L)** Same as in **(A–F)** but for units which preferred SF was outside 1 octave of the oddball SF. *p < 0.05, ***p < 0.001, ns = not significant.

To investigate whether adaptation and change detection depend on the tuning properties of the units, we split neurons based on their preferred SF. It was defined as a peak (maximum) of the fitted tuning curve of the unit. Based on preferred SF, we then split units into three groups: tuned_in group included neurons with preferred SF that was within ±1 octave of the oddball SF, 0.03 cpd (0.015 < pref SF < 0.06; Figure 4A, gray shaded region); tuned_out group included units with preferred SF that was outside the ± 1 octave of the oddball SF (pref SF < 0.015 or pref SF > 0.06; Figure 4G, gray shaded region); the untuned group included units that did not show any SF tuning, so that curve fitting was not successful or fitting error was larger than 0.9 (“Materials and Methods” section).

We first focused on oddball responses of tuned_in units (Figures 4A–F). iSSA and iMM modulation indices [−1, +1] quantify how strong a given unit is adapted and report MM response correspondingly (positive values indicate stronger modulation). We observed that the majority of tuned_in neurons show both SSA and MM in both genotypes [Figure 4B, note marginal distributions). Direct comparison of iSSA and iMM distributions did not reveal any differences between WT and FX mice (Figure 4C, WT vs. FX iSSA (*P* = 0.803) and iMM (*P* = 0.325), *n* = 201 and 147 units, Kolmogorov–Smirnov 2 sample test]. Unit population responses revealed an overall strong adaptation in both genotypes, which is not surprising given that the preferred SF of these units was close to the oddball SF. Interestingly, tuned_in units also show strong MM responses [Figure 4F, STD vs. CTR WT (*P* = 1.04e-10) and FX (*P* = 1.58e-7); STD vs. DEV WT (*P* = 0.0003) and FX (*P* = 0.0002), *n* = 249 and 184 units, Mann–Whitney *U* test]. This diverges from theories suggesting that enhancement of DEV response is primarily due to the non-adapted units in the local microcircuit (Ross and Hamm, 2020). The proportion of tuned_in units was comparable between genotypes (Supplementary Figure 2). Tuned_out units also showed both SSA and MM at the single-unit level (Figure 4H). Distribution of iMM but not iSSA was significantly different between groups [Figure 4I, WT vs. FX iSSA (*P* = 0.102) and iMM (*P* = 0.019), *n* = 235 and 193 units, Kolmogorov–Smirnov 2 sample test]. There was a significant adaptation at the population level in both genotypes, which suggests that adaptation spreads to the units preferring distant SFs (Figures 4J,K). Strong MM responses were also present in both genotypes [Figure 4L, STD vs. CTR WT (*P* = 9.04e-8) and FX (*P* = 0.014); STD vs. DEV WT (*P* = 2.58e-7) and FX (*P* = 0.0006), *n* = 341 and 278 units, Mann–Whitney *U* test].

### Altered Oddball Responses in Untuned and Inhibited Units of FX Mice

**Figure 5:**
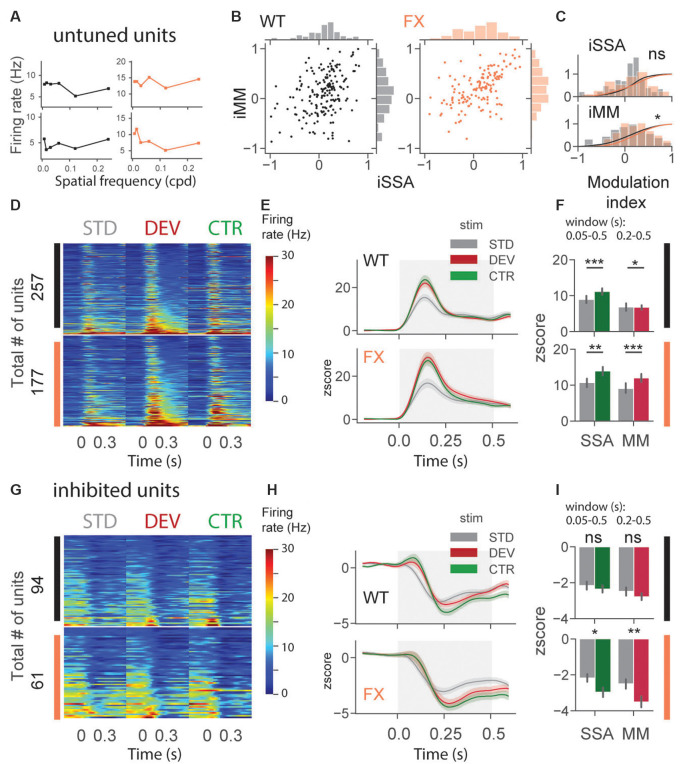
Altered oddball responses in untuned and inhibited units of FX mice. **(A)** Example SF tuning curves of untuned neurons. Two criteria were used to identify those units: (1) failure of DOG model fitting or (2) high fitting error (>0.9). **(B)** Distribution of iSSA and iMM modulation indices for WT and FX mice (each point is a single unit). **(C)** Superimposed distributions of iSSA and iMM with KDE. **(D)** The heatmaps show single-unit firing rates in response to STD, DEV, and CTR stimuli across different genotypes. **(E)** The line plots represent mean *z*-scored responses of the units from the heatmaps. **(F)** The point plots show the mean ± SEM of the *z*-scored firing rate between 0.05–0.5 s for SSA and 0.2–0.5 s for MM relative to the stimulus onset. **(G–I)** Same as in **(D–F)** but for inhibited units. *p < 0.05, **p < 0.01, ***p < 0.001, ns = not significant.

An identical analysis was performed for untuned and inhibited unit oddball responses (Figure 5). Untuned units are not tuned to a particular SF (Figure 5A), and the inhibited group was suppressed by visual stimuli. Oddball responses in the untuned group were diverse in both genotypes (Figure 5B). We found a significant difference in iMM distribution between genotypes [Figure 5C, WT vs. FX iSSA (*P* = 0.061) and iMM (*P* = 0.023), *n* = 178 and 145 units, Kolmogorov–Smirnov 2 sample test]. Unit population responses showed adaptation in both genotypes, whereas MM was not present in WT animals, the latter part of the STD response was slightly stronger than DEV [Figures 5D–F, STD vs. CTR WT (*P* = 1.52–5) and FX (*P* = 0.0011); STD vs. DEV WT (*P* = 0.023) and FX (*P* = 0.0003), *n* = 257 and 177 units, Mann–Whitney *U* test]. Interestingly, DEV and CTR evoked significantly stronger inhibition in FX, but not in WT mice [**Figures 5G–I**, STD vs. CTR WT (*P* = 0.226) and FX (*P* = 0.011); STD vs. DEV WT (*P* = 0.065) and FX (*P* = 0.005), *n* = 94 and 61 units, Mann–Whitney *U* test]. Contextual modulation of inhibited units in FX but not in WT mice might suggest an altered coupling of regular and fast-spiking (RS and FS) neurons.

### Adaptation Depends on the Spatial Frequency Tuning of the Units and Is Reduced in FX Animals

**Figure 6:**
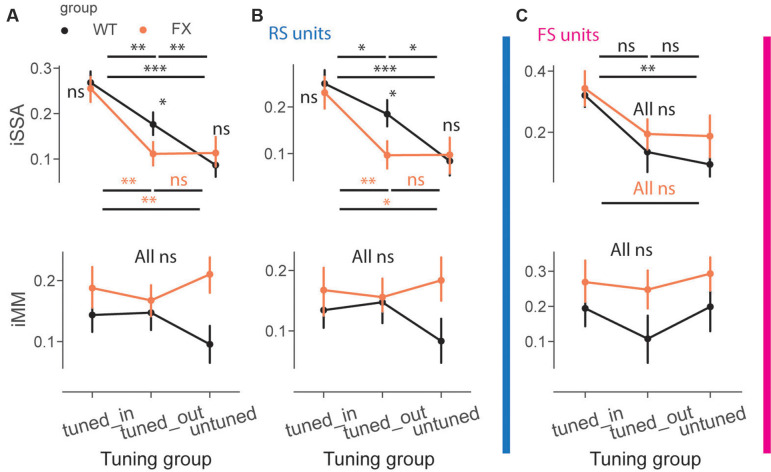
Adaptation depends on the preferred SF of the units. **(A)** The point plots show iSSA and iMM magnitude for tuned_in, tuned_out, and untuned group for WT and FX for all units. **(B)** Same as in **(A)**, but for RS units. **(C)** Same as in **(A)**, but for FS units. *p < 0.05, **p < 0.01, ***p < 0.001, ns = not significant.

We next directly compared iSSA and iMM magnitude across different tuning groups and genotypes (Figure 6A). First, we observed that iSSA was significantly larger in tuned_in compared to other groups in both genotypes. Interestingly, tuned_out units show stronger adaptation than untuned in WT, but not in FX animals. Furthermore, iSSA was significantly larger in WT vs. FX tuned_out units [Figure 6A top, iSSA: WT tuned_in vs. tuned_out (*P* = 0.005), tuned_in vs. untuned (*P* = 1.45e-8), tuned_out vs. untuned (*P* = 0.005); FX tuned_in vs. tuned_out (*P* = 0.0003), tuned_in vs. untuned (*P* = 0.002), tuned_out vs. untuned (*P* = 0.465); WT vs. FX tuned_in (*P* = 0.419), tuned_out (*P* = 0.041), and untuned (*P* = 0.252), *n* = 201, 235 and 178 WT units, 147, 193, and 145 FX units, Mann–Whitney *U* test, *p-values* were adjusted for multiple comparisons using the Benjamini–Hochberg method]. MM responses, on the other hand, were not significantly modulated by tuning properties of neurons [Figure 6A top, all comparisons (*P* > 0.05)]. We did not observe any systematic patterns between iSSA/iMM and preferred SF at the single unit level (Supplementary Figure 4).

It has been recently reported that FS neurons are differentially modulated in V1 of FX mice. Thus, we investigated whether oddball processing is altered in FS units (Supplementary Figure 3). SSA and MM responses were observed in FS of both genotypes (Supplementary Figure 3). We thus decided to investigate how iSSA and iMM are represented in RS and FS units. We observed that difference in RS rather than FS units mostly accounted for the differences observed across different tuning groups and genotypes [Figures 6B,C top, RS: WT tuned_in vs. tuned_out (*P* = 0.041), tuned_in vs. untuned (*P* = 7.0e-5), tuned_out vs. untuned (*P* = 0.013); FX tuned_in vs. tuned_out (*P* = 0.008), tuned_in vs. untuned (*P* = 0.011), tuned_out vs. untuned (*P* = 0.438); WT vs. FX tuned_in (*P* = 0.335), tuned_out (*P* = 0.036), and untuned (*P* = 0.461), *n* = 150, 175, and 141 WT units, 109, 148, and 101 FX units; **FS**: WT tuned_in vs. untuned (*P* = 0.003), all other comparisons (*P* > 0.05), Mann–Whitney *U* test, *p-values* were adjusted for multiple comparisons using the Benjamini–Hochberg method]. MM responses were not significantly modulated by tuning properties in RS and FS units [Figures 6B,C top, all comparisons (*P* > 0.05)]. The proportion of units in each subgroup was comparable between genotypes (Supplementary Figure 2). Overall, our results suggest that adaptation depends on the tuning properties of units but not their laminar position along with reduced feature co-adaptation in FX animals.

### Impaired Laminar Processing of MM Responses in FX Mice

**Figure 7:**
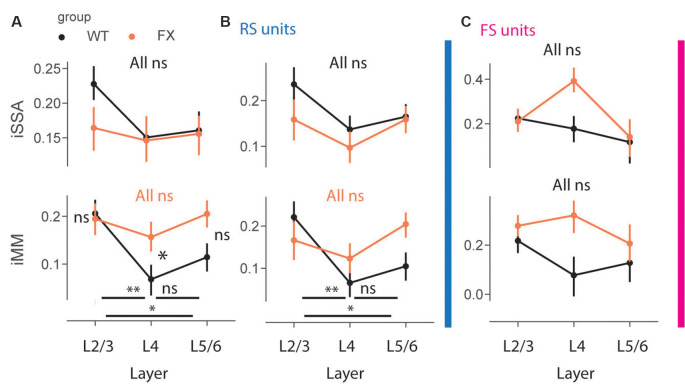
Impaired laminar processing of MM responses in FX mice. **(A)** The point plots show iSSA and iMM magnitude for L2/3, L4, and L5/6 for WT vs. FX for all units. **(B)** Same as in **(A)**, but for RS units. **(C)** Same as in **(A)**, but for FS units. *p < 0.05, **p < 0.01, ns = not significant.

To gain insight into laminar processing of oddball responses, we quantified population level iSSA and iMM modulation indices across different cortical layers (Figure 7). Adaptation was similarly represented across the cortical column in both genotypes, however, there was a trend towards stronger iSSA in superficial layers of WT mice [Figures 7A–C top, all comparisons (*P* > 0.05)]. iMM responses, on other hand, were significantly modulated by cortical layers. They were significantly stronger in L2/3 vs. L4 and L5/6 in WT, however, there was not any laminar preference for MM responses in FX mice. Furthermore, L4 MM responses were significantly stronger in FX vs. WT mice [Figure 7A top, iMM: WT tuned_in vs. tuned_out (*P* = 0.0018), tuned_in vs. untuned (*P* = 0.04), tuned_out vs. untuned (*P* = 0.242); FX tuned_in vs. tuned_out (*P* = 0.281), tuned_in vs. untuned (*P* = 0.431), tuned_out vs. untuned (*P* = 0.319); WT vs. FX tuned_in (*P* = 0.431), tuned_out (*P* = 0.042), and untuned (*P* = 0.068), *n* = 208, 191 and 215 WT units, 154, 153, and 1,178 FX units, Mann–Whitney *U* test, *p-values* were adjusted for multiple comparisons using the Benjamini–Hochberg method]. RS units showed similar oddball responses [Figure 7B, iMM RS: WT tuned_in vs. tuned_out (*P* = 0.005), tuned_in vs. untuned (*P* = 0.04), tuned_out vs. untuned (*P* = 0.237); FX tuned_in vs. tuned_out (*P* = 0.281), tuned_in vs. untuned (*P* = 0.321), tuned_out vs. untuned (*P* = 0.148); WT vs. FX tuned_in (*P* = 0.237), tuned_out (*P* = 0.189), and untuned (*P* = 0.085), *n* = 129, 154 and 183 WT units, 87, 122, and 149 FX units; **FS**: all comparisons (*P* > 0.05), Mann–Whitney *U* test, *p-values* were adjusted for multiple comparisons using the Benjamini–Hochberg method]. iSSA and iMM responses in FS units were not significantly modulated by cortical layers, though there was a trend towards stronger adaptation in L4 of FX mice (Figure 7C). It is unlikely that tuning properties of neurons can explain these observations because there is no difference in cortical distribution of different tuning groups between WT and FX animals (Supplementary Figure 5). Taken together, these findings suggest that there is a laminar specialization for MM responses in WT but not in FX animals.

### Altered Representation of Omission Responses in FX Mice

**Figure 8:**
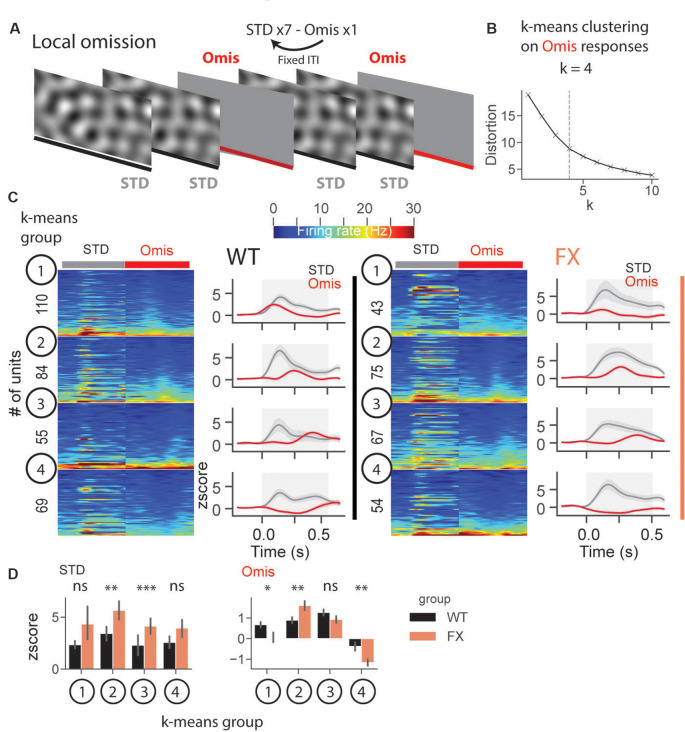
Altered representation of omission responses in FX mice. **(A)** During the omission paradigm every eighth stimulus was not presented (omission). **(B)** The number of groups for *k*-means was determined using the elbow method. Clustering was performed on omission responses (0.05–0.5 s) from units of both genotypes. Given that genotype is independent of clustering, we compared neural responses between WT and FX within each *k-means* group. **(C)** The heatmaps of unit firing rate responses across different *k-means* groups and genotypes (left = STD, right = Omis). The line plots show the mean *z* score firing rate responses of units shown in the heatmaps. 1st *k-means* group shows early, 2nd group mid, and 3rd group late omission responses, and 4th group was inhibited by Omis. **(D)** The point plots show the mean ± SEM *z* score firing rate for STD (left) and Omis (right) responses for WT and FX. *p < 0.05, **p < 0.01, ***p < 0.001, ns = not significant.

In a subset of animals, we performed omission experiments, in which every eighth stimulus was omitted (Figure 8A). Omission responsive neurons were defined as those with significantly different stimulus (0.05–0.35 s) vs. baseline (−0.25–0.05 s) responses (both excited and inhibited see Supplementary Figures 6B–D). Laminar analysis of omission responses did not reveal any differences between WT and FX mice (Supplementary Figure 6E). We then decided to use an unsupervised clustering algorithm, *k*-means, to reveal neural dynamics during omissions of the stimulus. Clustering was performed on neural responses within 0.05–0.5 s relative to the stimulus onset from both genotypes. Using an elbow method, we determined that *k* = 4 was an optimal number of groups (Figure 8B). Given that genotype was independent of clustering, we were able to compare responses between WT and FX within each *k*-means group. Clustering revealed four different types of responses: *k-means* group 1—early, group 2—mid, group 3—late omission responses, and group 4 was inhibited by the omission [Figure 8C). Direct comparison of STD between WT and FX revealed stronger responses in FX groups 2 (mid) and 3 (late), which might indicate reduced adaptation during the omission paradigm. Omission responses were stronger in *k-means* group 1 (early) in WT, whereas group 2 (mid), and group 4 (inhibited) were stronger in FX mice [Figure 8D, WT vs. FX *k-means* group 1 STD (*P* = 0.436), *n* = 110 and 43 units, Omis (*P* = 0.042), *n* = 120 and 45 units; group 2 STD (*P* = 0.004), *n* = 84 and 75 units, Omis (*P* = 0.009), *n* = 85 and 77 units; group 3 STD (*P* = 0.0001), *n* = 55 and 67 units, Omis (*P* = 0.052), *n* = 58 and 70 units, group 4 STD (*P* = 0.200), *n* = 69 and 54 units, Omis (*P* = 0.005), *n* = 73 and 59 units, Mann–Whitney *U* test]. Overall, we found the altered processing of omission responses in FX animals.

## Discussion

The lack of a common framework to explain the disparate sensory and social-cognitive deficits in FX and autism is a major roadblock to scientific progress and designing effective diagnostic and intervention tools. Atypical sensory processing has recently been recognized to be an important diagnostic criterion for autism (American Psychiatric Association, 2013). Furthermore, early sensory alterations are predictive of social communication deficits later in life (Robertson and Baron-Cohen, 2017). Investigating the reproducible sensory perception paradigms in well-defined genetic models of autism provides a great opportunity to shed light on the neural basis of atypical sensory experience and its possible interaction with social-cognitive domains in ASD.

Here, we used a novel visual oddball paradigm and silicon probe recordings in V1 to investigate the neural basis of altered sensory perception in FX. Using SF tuning, we first demonstrated that high SF bands are excessively processed in the late stages of visual responses in FX mice. Increased firing rate and lower SF decoding errors at late stages of processing are indicative of over-processing of details. This finding is consistent with previous psychophysical and physiology studies showing altered spatiotemporal processing of high SF information in autism (Kéïta et al., 2014; Caplette et al., 2016). Interestingly, we didn’t observe any difference in SF tuning between genotypes while focusing on peak responses.

Using SF oddball paradigm, we then showed that there was a differential contextual processing in V1 of FX mice across different cortical layers and unit types (Table 1). To investigate the feature specificity of SSA and MM responses, we split neurons into three groups based on their SF preference. We discovered that adaptation was more dependent on the tuning preferences rather than the laminar position of the units. SSA was strongest in tuned_in units in both genotypes, which is not surprising given that their preferred SF was close to the oddball SF (Chen et al., 2015). We observed comparable adaptation levels in tuned_in and tuned_out group in WT but not in FX animals (Table 1). Interestingly, RS but not FS units were mostly responsible for the observed differences. Analysis of SSA across different cortical layers revealed the strongest adaptation in L2/3 in WT, but it did not reach significant after adjustment for multiple comparisons. Overall, SSA was dependent on the preferred SF of the units and covered a narrower range of spatial frequencies in FX compared to WT animals. This observation might be explained by the reduced spread of adaptation (co-adaptation to neighboring SF) in FX. Our results may provide a mechanism for the reduced habituation and sensory hypersensitivity in FX and autism.

**Table 1:** Summary of differences in oddball and omission responses between WT and FX animals.

Oddball/pref SF	Tuned_in	Tuned_out	Untuned
iSSA	ns	↑*	ns
iMM	ns	ns	ns
Oddball/Layer	L2/3	L4	L5/6
iSSA	ns	ns	ns
iMM	ns	↓*	ns
Omission/*k*-means	Group 1	Group 2	Group 3	Group 4
STD	ns	↓**	↓***	ns
Omis	↑*	↓**	ns	↓**

↑ stronger in WT and weaker in FX; ↓ weaker in WT and stronger in FX; *p < 0.05, **p < 0.01, ***p < 0.001, ns = not significant.

Mismatch responses, on the other hand, were more dependent on the laminar position rather than the tuning preference of units. MM responses were present in the adapted units, suggesting that single units might report mismatch despite strong adaptation levels (Ross and Hamm, 2020). L2/3 had the strongest MM responses in WT, but not in FX, where they were equally represented across the cortical column. Furthermore, L4 MM responses were significantly stronger in FX mice (Table 1). These observations might be explained by the altered intrinsic properties of L4 neurons similar to the previously reported observations in the somatosensory cortex (Booker et al., 2019). The lack of laminar specialization for MM in FX might also be linked to the altered information processing in L4 barrel cortex (Domanski et al., 2019). It is important to note that RS units were mostly responsible for the observed differences in MM. This observation is consistent with the previous studies of the reduced excitatory drive onto FS units, which may potentially explain the altered dynamics of FS interneurons (Gibson et al., 2008; Goel et al., 2018).

Lastly, we observed the altered neural dynamics in FX animals during the omission paradigm. Interestingly, STD responses were weaker in WT vs. FX animals, which might be indicative of reduced adaptation in FX animals. Our unsupervised clustering revealed four different types of responses to stimulus omissions. Interestingly, these groups had different temporal patterns covering the whole omission duration with early, mid, late peak responses and inhibition. Early omission responses were stronger in WT, whereas mid and inhibition ones were enhanced in FX animals. We also observed increased delayed responses during SF tuning, oddball, and omission paradigms, which suggests that it might be a common pattern in FX circuits (Table 1). Given the regularity of omission responses (every eighth stimulus) and fixed inter-trial-interval, we expected the animals to be entrained by the sequence. Overall, reduced STD responses and stimulus timing-locked omission responses suggest that WT but not FX animals were able to learn the regularity of the sequence of stimuli.

In conclusion, we extend prior oddball studies by showing how tuning properties, laminar position, and spiking profile of the neurons influence the contextual processing of visual information. Our discovery of reduced adaptation and altered laminar processing in FX mice provides the mechanistic circuit-level understanding of the impaired sensory perception in FX and might lead to potential diagnostic and therapeutic advances.

## Data Availability Statement

The raw data supporting the conclusions of this article will be made available by the authors, without undue reservation.

## Ethics Statement

The animal study was reviewed and approved by PACUC.

## Author Contributions

AP and AC designed the study. AP and SK performed the experiments. AP analyzed the data. AP, SK, and AC wrote the manuscript. All authors contributed to the article and approved the submitted version.

## Conflict of Interest

The authors declare that the research was conducted in the absence of any commercial or financial relationships that could be construed as a potential conflict of interest.
