# Supplementary material for: Impaired Adaptation and Laminar Processing of the Oddball Paradigm in the Primary Visual Cortex of Fmr1 KO Mouse
Source: Front Cell Neurosci. 2021 May 19;15:668230. doi: 10.3389/fncel.2021.668230 (PMC8170411; doi:10.3389/fncel.2021.668230)
Supplement: Supplementary file 1 [file Image_1.pdf]

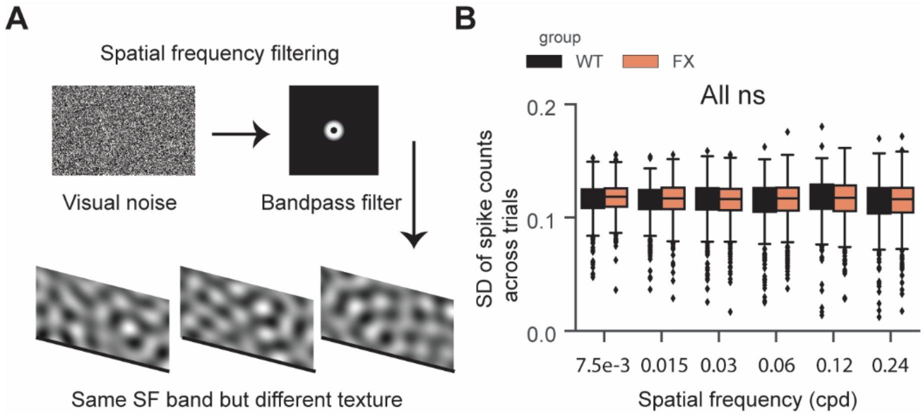

Supplementary Figure 1. Stimuli of the same SF band but different overall patterns induce similar neural responses. **A.** Spatial frequency filtering generates stimuli with the same SF band but different global pattern **B.** Box plots show neural variability (SD of spike counts across trials) for each SF stimulus used in SF tuning experiment (WT vs FX: SF7.5e-3 ($P = 0.14$), SF0.015 ($P = 0.16$), SF0.03 ($P = 0.29$), SF0.06 ($P = 0.32$), SF0.12 ($P = 0.16$), and SF0.24 ($P = 0.29$), n = 594 and 562 units, Mann-Whitney U test, p-values were adjusted for multiple comparisons using Benjamini-Hochberg method).

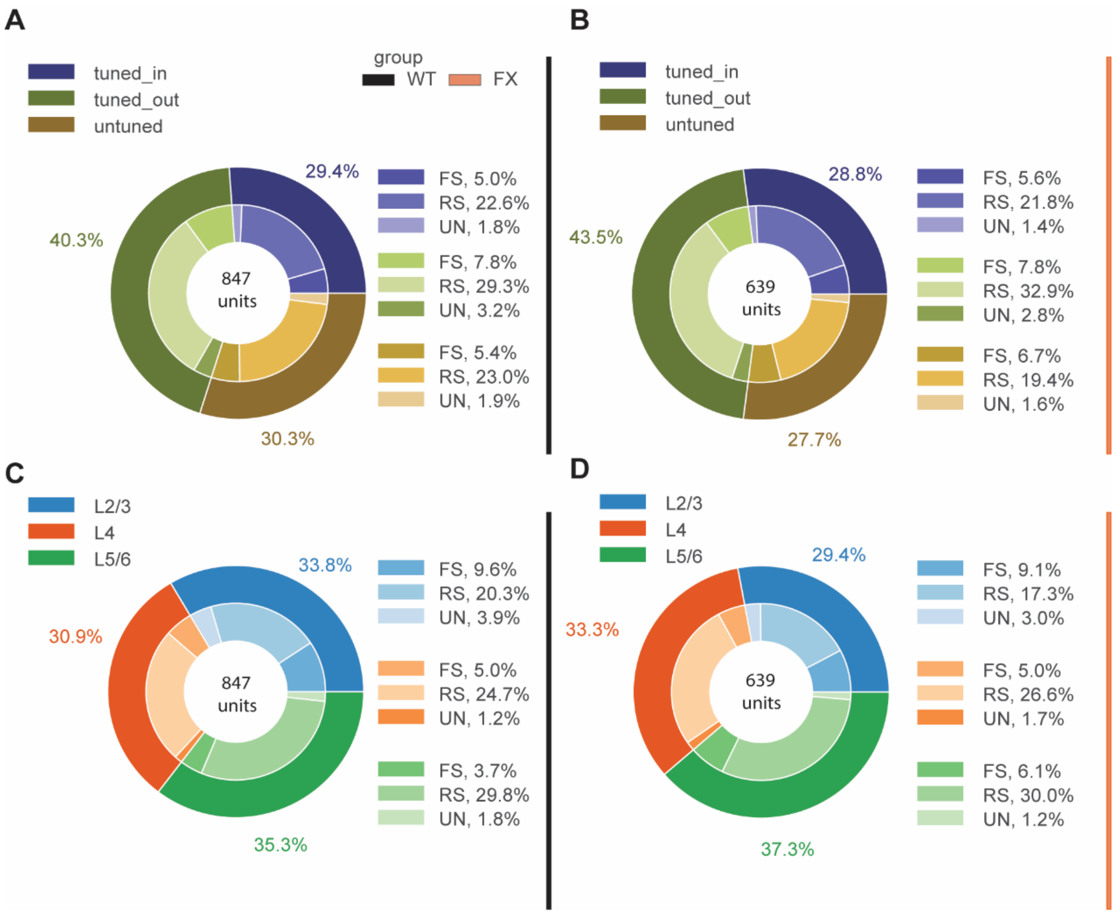

Supplementary Figure 2. A similar representation of different types and groups of neurons in WT and FX mice. **A.** The nested pie chart shows the proportion of units grouped by tuning preference and spiking profile for WT mice. The outer pie chart shows the percentage of units in tuned_in, tuned_out, and untuned group. The inner chart shows the percentage of regular spiking, fast spiking, and unclassified neurons in each subgroup. **B.** Same as in A, but for FX animals. **C.** The nested pie chart shows the proportion of units across the cortical layer for WT mice. The outer pie chart shows the percentage of neurons in layer 2/3, layer 4, and layer 5/6. The inner chart shows the percentage of regular spiking, fast spiking, and unclassified neurons in each subgroup. **D.** Same as in C, but for FX mice.

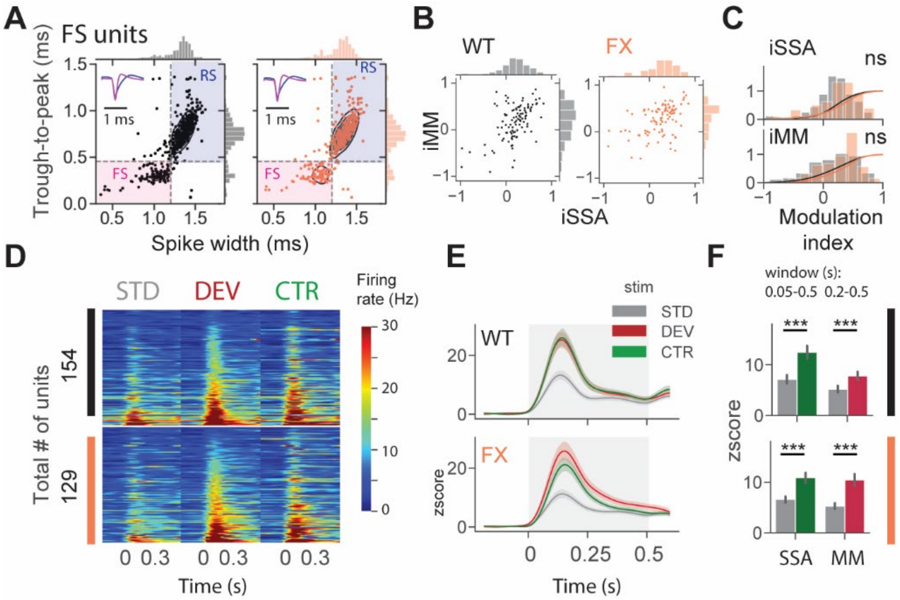

Supplementary Figure 3. SSA and MM are present in fast spiking units of both genotypes. **A.** Units were split into regular, fast spiking, and unclassified based on their template waveform features such as spike width (spw) and trough-to-peak (t-p) time. Fast spiking units had shorter spike width, trough-to-peak time, and narrow waveform (WT and FX spw (0.97 and 1.01 ms), t-p (0.30 and 0.30 ms)). Regular spiking units, on the other hand, had broader waveforms (WT and FX spw (1.36 and 1.37 ms), t-p (0.80 and 0.80 ms)). **B.** Distribution of iSSA and iMM modulation indices for WT and FX mice (each point is a single unit). **C.** Superimposed distributions of iSSA and iMM with KDE (WT vs FX iSSA (P = 0.086) and iMM (P = 0.071), n = 105 and 100 units, Kolmogorov-Smirnov 2 sample test). **D.** The heatmaps show single-unit firing rates in response to STD, DEV, and CTR stimuli across different genotypes. **E**. The line plots represent the mean z-scored responses of the units from the heatmaps. **F.** The point plots show the mean ± s.e.m. of the z-scored firing rate between 0.05-0.5s for SSA and 0.2-0.5s for MM relative to the stimulus onset (STD vs CTR WT (P = 0.0001) and FX (P = 0.0005); STD vs DEV WT (P = 0.0009) and FX (P = 4.31e-5), n = 154 and 129 units, Mann-Whitney U test).

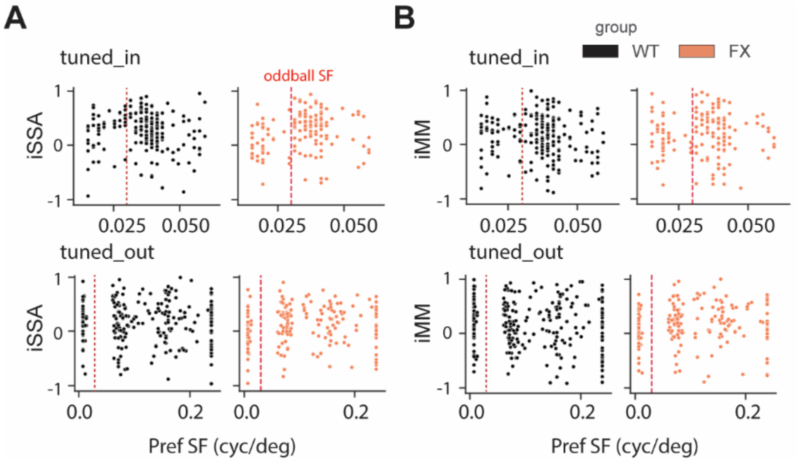

Supplementary Figure 4. Oddball responses are modulated by the preferred SF of the units. **A.** The scatter plots show the relationship between iSSA vs. preferred SF of the units from tuned_in (top) and tuned_out (bot) group for WT and FX mice. Each dot represents a single unit. The vertical dotted line represents the oddball SF. **B.** Same as in A but for iMM vs. preferred SF of the units.

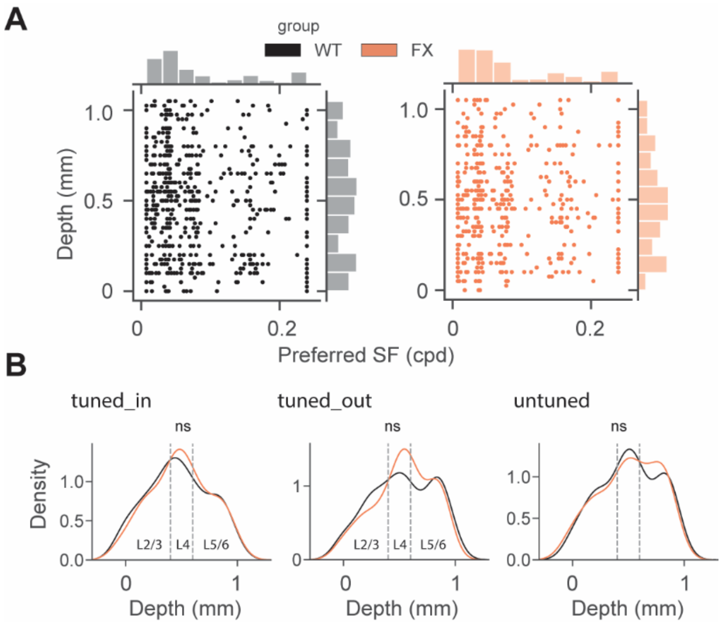

Supplementary Figure 5. The cortical distributions of the tuned_in, tuned_out, and untuned groups are not different between genotypes. **A.** The scatter plots show the relationship between cortical depth and preferred SF of the units from WT and FX mice. Each dot represents a single unit. **B.** Cortical distribution of units for each tuning group for WT and FX mice (WT vs. FX tun_in (P = 0.766), n = 249 and 184 units; tuned_out (P = 0.253), n = 341 and 278; untuned (P = 0.852), n = 257 and 177 units, Kolmogorov-Smirnov 2 sample test).

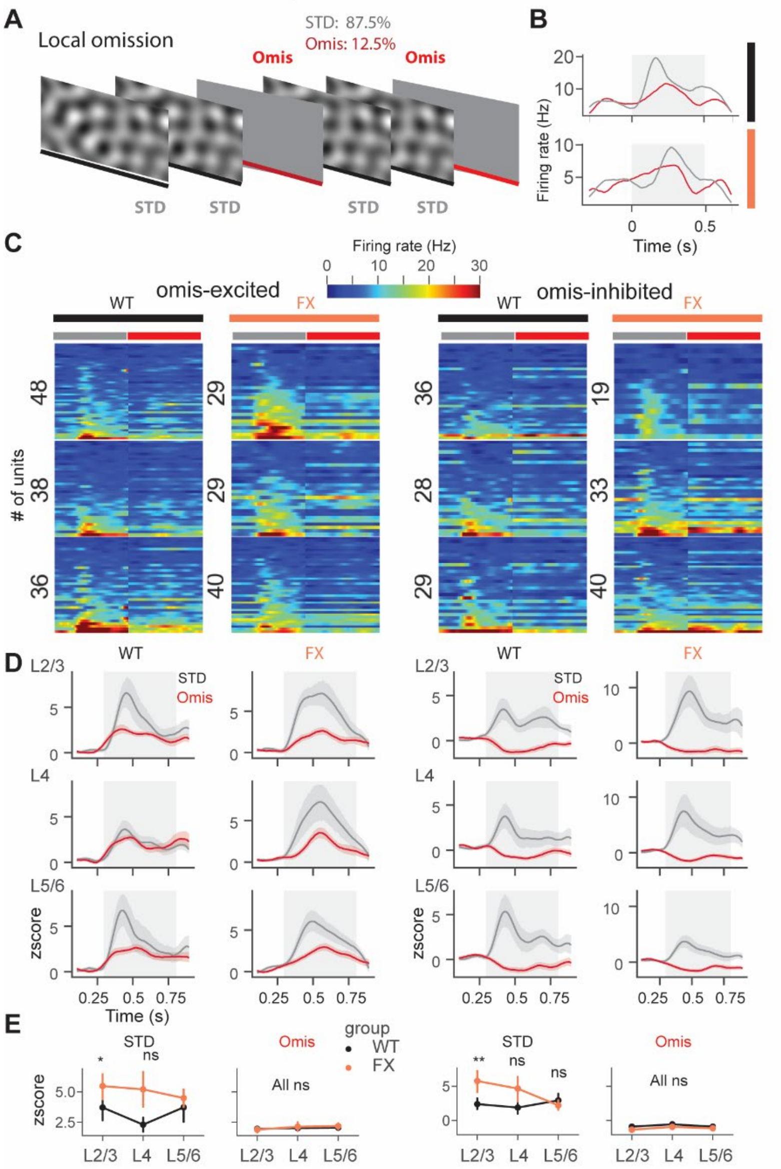

Supplementary Figure 6. Neural responses to stimulus omissions are present across the cortical column in both WT and FX mice. **A.** During the omission paradigm every eighth stimulus was not presented (omission). **B.** Example unit responses to the STD and Omis in WT and FX mice. **C.** The heatmaps of unit firing rate responses across different cortical layers, response types, and genotypes (left = STD, right = Omis). The first two columns show units, which were excited by the omission, whereas two columns on the right show units that were inhibited by the omission of the stimuli. **D**. The line plots show the mean z-score firing rate responses of units shown in the heatmaps for excited (left) and inhibited (right) population. **E**. Point plots show the mean ± s.e.m. z-scored firing rate of excited (left two) and inhibited (right two) population across different layers for WT vs FX (**Omis-excited STD:** WT vs FX L2/3 ($P = 0.021$), n = 48 and 29 units, L4 ($P = 0.193$), n = 38 and 29, L5/6 ($P = 0.073$), n = 36 and 40 units; **Omis:** all comparisons ($P > 0.05$); **Omis-inhibited STD:** WT vs FX L2/3 ($P = 0.009$), n = 36 and 19 units, L4 ($P = 0.074$), n = 28 and 33, L5/6 ($P = 0.306$), n = 29 and 40 units; **Omis:** all comparisons ($P > 0.05$); Mann-Whitney U test).
